# Supplementary material for: Differential weighting of information during aloud and silent reading: Evidence from representational similarity analysis of fMRI data
Source: Imaging Neurosci (Camb). 2025 Jan 13;3:imag_a_00428. doi: 10.1162/imag_a_00428 (PMC12319848; doi:10.1162/imag_a_00428)
Supplement: Supplementary Material [file imag_a_00428-supp.pdf]

Supplementary Materials

Details of dissimilarity measures used for construction of hypothesis models

For our visual measure, we first created an image of each word as it was presented in the experiment using the Pillow package (Umesh, 2012). Each image was then binarized (0 for background pixels, 1 for text pixels) and converted to a vector; we computed visual dissimilarity as the Pearson correlation distance between these vectors (as per Kriegeskorte et al., 2008). For orthography, we took a similar approach to Fischer-Baum et al., (2017): features were unconstrained open character bigrams, computed using the wordkit package (Tulkens et al., 2018), and dissimilarity was calculated as Pearson correlation distance between feature vectors.

For phonology, we used the method described by Bartelds et al. (2020), which is summarized in the manuscript proper. To this end, we collected voice samples from seven volunteers of similar demographic characteristics to our main study sample (three female, two male, one non-binary, age range 20-34)[1]. All volunteers reported that they were born and raised in North America (mostly Canada; one volunteer was from the US) and that English was their dominant language. We reasoned that these volunteers' speech patterns ought to be very similar to those of our fMRI participants, considering that our study took place in Canada. Each volunteer recorded voice samples in a small noise-shielded booth at Dalhousie University. Voice samples were recorded using *Audacity* (version 3.5; https://www.audacityteam.org/) operating on a laptop computer connected to a Logitech YETI USB microphone. Each volunteer was asked to read the full list of study words (S Table 1) aloud in a neutral voice, in alphabetical order. Each volunteer read the full list of words twice, thus providing two samples for every word in the list. In addition to these recordings, we also obtained AI-generated voice samples using the text-to-speech tool provided by the online platform *Vecticon* (https://vecticon.co). We generated speech samples using four of *Vecticon*'s built-in voices: Clara (female, Canadian), Liam (male, Canadian), Jenny (female, American), Davis (male, American). We selected these voices

[1] All volunteers gave permission for their voice samples to be used for analysis purposes, and for their anonymized recordings to be made publicly available in our online data repository.

because they all simulate adult speakers with North American accents, and therefore (as with our volunteers) should approximately reflect the speech patterns of our participants.

For semantic models, we used the the gensim package (Řehůřek & Sojka, 2010) to extract vectors from the publicly available glove-wiki-gigaword-300 model (https://github.com/RaRe-Technologies/gensim-data); a pre-trained model of natural language processing based on the Global Vectors for Word Representation algorithm (GLoVe; Pennington et al., 2014). We computed semantic dissimilarity as the cosine distance between vectors, consistent with prior RSA work using so-called word2vec semantic models (e.g. Carota et al., 2021; Guo et al., 2022; Liu et al., 2021; Tong et al., 2022; Wang et al., 2018).

Articulatory models were created using the PhonologicalCorpusTools package (Hall et al., 2019) in conjunction with the Irvine Phonotactic Online Dictionary (IPHOD; Vaden et al., 2009). Each word was transcribed into a string of its constituent phonemes; in turn, each phoneme was represented by a set of features as defined by Hayes (2008). Features reflected actions of the mouth, tongue, and vocal tract required to produce that phoneme (e.g., a phoneme may be voiced or voiceless, has a particular place of articulation, entails some degree of constriction by the lips, teeth, and vocal tract, etc.); thus, we considered them to reflect articulatory components of speech. Articulatory dissimilarity was computed as the edit distance between transcribed phoneme strings (that is, the number of additions, substitutions and/or deletions required to convert one phoneme string to another), weighted according to the number of features shared between constituent phonemes (Fontan et al., 2016). To control for confounding effects of word length, pairwise dissimilarity values were normalized by dividing the computed edit distance by the length of the longest word in the pair (as per Beijering et al., 2008; Schepens et al., 2012).

Details of measures for extraneous properties

As described in the manuscript proper, we computed measures of the following uncontrolled properties: imageability, morphological consistency, concreteness, grapheme-to-phoneme consistency, and syntactic category. Numerical imageability and

concreteness ratings for each word were obtained from Scott et al. (2019) and Brysbaert et al., (2014) respectively, and dissimilarity between words was computed as the absolute numerical difference between words in both cases. For morphological complexity, we counted the number of morphemes in each word; dissimilarity was computed as the absolute difference in morpheme counts. For grapheme-to-phoneme consistency, words were vectorised according to their syllable-wise spelling-to-sound consistency (Chee et al., 2020) and dissimilarity was computed as the Euclidean distance between vectors (we found that using Euclidean distances produced overall more normally distributed hypothesis models, compared with other metrics such as correlation or cosine distance). For syntactic category, each word was assigned a binary value: 0 if the word was a noun only, 1 if the word was both a noun and a verb. Dissimilarity was again computed as the absolute difference in value.

Supplementary tables

S Table 1. List of words used in the current study

account	century	garden	language	pebble	summer
answer	department	handle	machine	powder	ticket
author	education	industry	message	record	turnip
beauty	envelope	journey	ocean	sailor	valley
campaign	forest	kingdom	painting	speech	wheat

S Table 2. Values show correlations between hypothesis models (used for RSA) and potentially confounding stimulus properties. Parenthetical values are Bayes factors indicating strength of evidence for each correlation. Abbreviations: G2P consistency = grapheme-to-phoneme consistency; Morph. complexity = morphological complexity.

	**Visual**	**Orthographic**	**Phonological**	**Semantic**	**Articulatory**
**Concreteness**	0.06 (0.09)	-0.01 (0.04)	0.11 (0.61)	0.15 (6.03)	0.03 (0.05)
**G2P consistency**	-0.04 (0.06)	-0.03 (0.05)	0.22 (2072.86)	-0.14 (3.38)	0.12 (0.79)
**Imageability**	-0.02 (0.06)	-0.06 (0.07)	0.21 (7.35)	0.15 (0.82)	0.11 (0.21)
**Morph. complexity**	0.09 (0.22)	< 0.01 (0.04)	0.14 (3.43)	0.02 (0.04)	-0.05 (0.07)
**Syntactic category**	-0.04 (0.06)	-0.07 (0.11)	-0.04 (0.05)	< -0.01 (0.04)	-0.06 (0.08)

Supplementary References

Bartelds, M., Richter, C., Liberman, M., & Wieling, M. (2020). A New Acoustic-Based Pronunciation Distance Measure. *Frontiers in Artificial Intelligence*, *3*. https://doi.org/10.3389/frai.2020.00039

Beijering, K., Gooskens, C., & Heeringa, W. (2008). Predicting intelligibility and perceived linguistic distance by means of the Levenshtein algorithm. *Linguistics in the Netherlands*, *25*(1), 13–24.

Brysbaert, M., Warriner, A. B., & Kuperman, V. (2014). Concreteness ratings for 40 thousand generally known English word lemmas. *Behavior Research Methods*, *46*(3), 904–911.

https://doi.org/10.3758/s13428-013-0403-5

Carota, F., Nili, H., Pulvermüller, F., & Kriegeskorte, N. (2021). Distinct fronto-temporal substrates of distributional and taxonomic similarity among words: Evidence from RSA of BOLD signals. *NeuroImage*, *224*, 117408. https://doi.org/10.1016/j.neuroimage.2020.117408

Chee, Q. W., Chow, K. J., Yap, M. J., & Goh, W. D. (2020). Consistency norms for 37,677 english words. *Behavior Research Methods*, *52*(6), 2535–2555. https://doi.org/10.3758/s13428-020-01391-7

Fontan, L., Ferrané, I., Farinas, J., Pinquier, J., & Aumont, X. (2016). Using phonologically weighted Levenshtein distances for the prediction of microscopic intelligibility. *Annual Conference Interspeech (INTERSPEECH 2016)*, 650–654.

Guo, W., Geng, S., Cao, M., & Feng, J. (2022). Functional Gradient of the Fusiform Cortex for Chinese Character Recognition. *eNeuro*, *9*(3). https://doi.org/10.1523/ENEURO.0495-21.2022

Hall, K. C., Mackie, J. S., & Lo, R. Y.-H. (2019). Phonological CorpusTools: Software for doing phonological analysis on transcribed corpora. *International Journal of Corpus Linguistics*, *24*(4), 522–535. https://doi.org/10.1075/ijcl.18009.hal

Hayes, B. (2008). *Introductory phonology* (Vol. 7). John Wiley & Sons.

Kriegeskorte, N., Mur, M., & Bandettini, P. (2008). Representational similarity analysis—Connecting the branches of systems neuroscience. *Frontiers in Systems Neuroscience*, *2*. https://www.frontiersin.org/articles/10.3389/neuro.06.004.2008

Liu, J., Zhang, H., Yu, T., Ren, L., Ni, D., Yang, Q., Lu, B., Zhang, L., Axmacher, N., & Xue, G. (2021). Transformative neural representations support long-term episodic memory. *Science Advances*, *7*(41), eabg9715. https://doi.org/10.1126/sciadv.abg9715

Pennington, J., Socher, R., & Manning, C. D. (2014). Glove: Global vectors for word representation. *Proceedings of the 2014 Conference on Empirical Methods in Natural Language Processing (EMNLP)*, 1532–1543.

Řehůřek, R., & Sojka, P. (2010). Software Framework for Topic Modelling with Large Corpora. *Proceedings of the LREC 2010 Workshop on New Challenges for NLP Frameworks*, 45–50.

Schepens, J., Dijkstra, T., & Grootjen, F. (2012). Distributions of cognates in Europe as based on Levenshtein distance*. *Bilingualism: Language and Cognition*, *15*(1), 157–166. https://doi.org/10.1017/S1366728910000623

Scott, G. G., Keitel, A., Becirspahic, M., Yao, B., & Sereno, S. C. (2019). The Glasgow Norms: Ratings of 5,500 words on nine scales. *Behavior Research Methods*, *51*(3), 1258–1270. https://doi.org/10.3758/s13428-018-1099-3

Tong, J., Binder, J. R., Humphries, C., Mazurchuk, S., Conant, L. L., & Fernandino, L. (2022). A Distributed Network for Multimodal Experiential Representation of Concepts. *Journal of Neuroscience*, *42*(37), 7121–7130. https://doi.org/10.1523/JNEUROSCI.1243-21.2022

Vaden, K. I., Halpin, H. R., & Hickok, G. S. (2009). *Irvine phonotactic online dictionary, Version 2.0.[Data file]*.

Wang, X., Xu, Y., Wang, Y., Zeng, Y., Zhang, J., Ling, Z., & Bi, Y. (2018). Representational similarity analysis reveals task-dependent semantic influence of the visual word form area. *Scientific Reports*, *8*(1), Article 1. https://doi.org/10.1038/s41598-018-21062-0
